# Public health round-up

**DOI:** 10.2471/BLT.25.010425

**Published:** 2025-04-01

**Forced population displacement in Sudan**

A sandstorm in a refugee camp in Adre, Chad. According to a 10 March public health situation analysis published by WHO, the Sudanese population is facing the world’s largest and fastest-growing displacement crisis, with an estimated 12.8 million people forcibly displaced.

**Figure:**
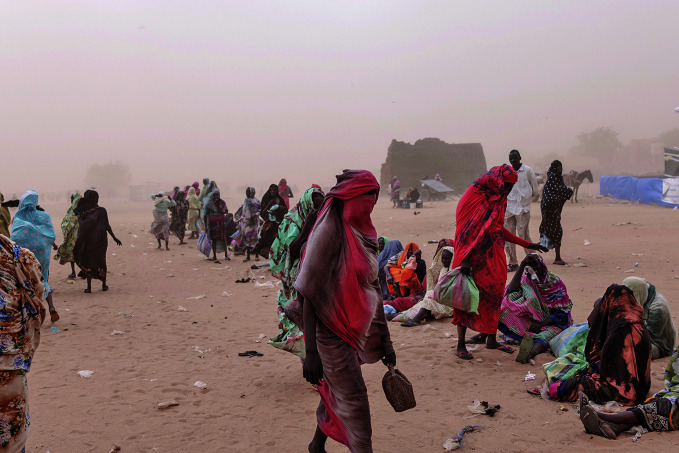
WHO/Nicolò Filippo Rosso

## Sudan virus disease 

As of 5 March 2025, a total of 14 people were reported to have been infected with Sudan virus disease (SVD) in Uganda. According to the World Health Organization (WHO) disease outbreak news published 8 March, 12 of those people had confirmed infections while two were assessed to be probably infected. Four of the people reported to have been infected (two confirmed and two probable) had died. These deaths included a child who was admitted to the Mulago hospital in Kampala on 23 February.

Eight of the people with confirmed infections received care at treatment centres in the capital Kampala and in the city of Mbale and were discharged on 18 February. As of 5 March, 192 people known to have been in contact with those diagnosed with SVD were being monitored and supported.

The Sudan virus species is distinct from the Zaire strain of Ebola virus, and its transmission cannot be prevented by the two vaccines licensed against the latter. However, a trial of a candidate vaccine for SVD was launched just four days after the outbreak was declared and a therapeutics trial was scheduled to start as soon as national authorities provided approval.

https://bit.ly/3XMdpiM

## Sudan crisis

Nearly two years into the conflict that has riven Sudan, the population is facing the world’s largest and fastest-growing displacement crisis, with an estimated 12.8 million people forcibly displaced.

According to a public health situation analysis published by WHO on 10 March, some 30.4 million people – over half the population – require assistance, including 16 million children.

Famine has been confirmed in multiple areas of North Darfur, while malnutrition rates are among the highest globally, with 4.9 million children under 5 years of age and pregnant women acutely malnourished.

Attacks on health care are compounding the challenges faced, with hundreds of incidents recorded, severely limiting access to medical care. Infectious diseases, including cholera, malaria and measles, are spreading.

WHO is supporting emergency response efforts, but insecurity, displacement and resource shortages continue to hinder health-care delivery across the country.

https://bit.ly/4kNKKDT

## Marburg virus disease

The Ministry of Health of the United Republic of Tanzania declared the end of the Marburg virus disease (MVD) outbreak. The 13 March declaration came after two consecutive incubation periods (a total of 42 days) since the last person confirmed with MVD died on 28 January 2025.

The outbreak was declared on 20 January 2025. As of 12 March 2025, 10 people were reported to have been infected (two confirmed and eight probable). All 10 died, including eight who had died prior to the confirmation of the outbreak. A total of 272 contacts that were listed for monitoring had completed their 21-day follow-up as of 10 February 2025.

https://bit.ly/41Q8iiO

## Funding cuts threaten tuberculosis response

Abrupt funding cuts to national tuberculosis (TB) programmes by the United States of America (USA) threaten to undo recent gains and put millions of people at grave risk.

This is according to a 5 March media release by WHO, which states that prior to the cuts, the USA government had been providing approximately 200–250 million United States dollars (US$) annually in bilateral funding for tuberculosis response at country level, roughly one quarter of the total amount of international donor funding.

According to WHO, the loss of this funding puts 18 of the highest tuberculosis burden countries at risk, with the WHO African Region hardest hit, followed by the WHO South-East Asia and Western Pacific Regions.

“Without immediate action, hard-won progress in the fight against TB is at risk.” said Dr Tereza Kasaeva, director of WHO’s Global Programme on TB and Lung Health. “Our collective response must be swift, strategic and fully resourced to protect the most vulnerable and maintain momentum towards ending TB.”

In the past two decades, tuberculosis prevention, testing and treatment services have saved more than 79 million lives – averting approximately 3.65 million deaths last year alone.

https://bit.ly/3FqVzf8

## Measles surges in the European Region

Some 127 350 measles cases were reported in the European Region for 2024, double the number reported for 2023 and the highest number since 1997.

This is according to a 13 March joint statement by WHO and the United Nations Children’s Fund. Citing preliminary data received as of 6 March 2025, the statement reports that children under 5 years of age accounted for more than 40% of the cases and that more than half of the total reported cases required hospitalization. A reported 38 measles patients had died. 

Measles cases in the Region have generally been declining since 1997, reaching a low of 4440 cases in 2016, followed by a resurgence in 2018 and 2019. A fall in immunization coverage during the coronavirus disease 2019 (COVID-19) pandemic was followed by a further rise in cases in 2023 and 2024. Vaccination rates in many countries have yet to return to pre-pandemic levels, increasing the risk of outbreaks.

“Measles is back, and it’s a wake-up call. Without high vaccination rates, there is no health security.” warned Dr Hans Henri P. Kluge, WHO Regional Director for Europe.

https://bit.ly/4kQn2qz

## Leading causes of maternal death

Haemorrhage and pre-eclampsia are the leading causes of maternal deaths globally. This is according to a new study published by WHO, which reveals that the conditions were responsible for around 80 000 and 50 000 fatalities, respectively, in 2020 – the last year for which published estimates are available – highlighting the fact that many women still lack access to lifesaving treatments and effective care during and after pregnancy and birth.

Published in *Lancet Global Health* on 8 March, the study is WHO’s first global update on the causes of maternal deaths since the United Nations sustainable development goals were adopted in 2015.

https://bit.ly/41EsGDq

## Preventing hearing loss among gamers

WHO and the International Telecommunication Union (ITU) released the first global standard for safe listening in video game and e-sport activities.

An estimated 3 billion people currently play video games on devices such as personal computers, video game consoles and mobile phones. Most devices and games lack safe listening features to protect users from harmful noise, exposing gamers to the risk of permanent hearing loss.

The WHO–ITU global standard is designed to protect hearing for all types of video game players, across a wide range of scenarios and equipment, and provides separate guidelines for devices and software.

https://bit.ly/41JhYeZ

## Substances placed under international control

On the recommendation of the WHO Expert Committee on Drug Dependence, the United Nations Commission on Narcotic Drugs placed five new psychoactive substances and one medicine under international control, the aim being to curb their misuse and protect public health.

Four synthetic opioids were classed as schedule I substances (substances with no known medical use, and associated with serious harm, including fatalities). A semi-synthetic cannabinoid was classed as a schedule II substance (substances with some recognized medical or therapeutic uses and a moderate to high risk of abuse and dependence); and a muscle relaxant was classed as a schedule IV substance (substances judged to have a lower potential for abuse compared to those in Schedules I, II and III, but still posing risks to public health).

“These substances have been flagged for being clandestinely manufactured, posing serious risks to public health and society,” said Dr Deus Mubangizi, WHO director for Health Product Policy and Standards, urging countries to take the actions needed to protect vulnerable populations.

https://bit.ly/4kOcCrw

**Cover photo**

Health workers welcome the arrival of cholera kits in Angola, where there have been outbreaks of the disease in Luanda, Icolo e Benog and Bengo provinces.

**Figure:**
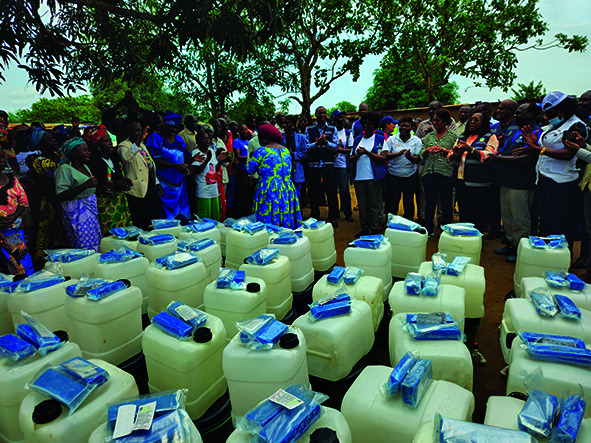
(WHO/Omotola Akindipe)

## Influenza vaccine composition

WHO announced its recommendations for the influenza vaccine composition for the 2025–2026 northern hemisphere influenza season. The 28 February announcement followed a four-day biannual meeting on influenza virus vaccine composition. The recommendations guide national regulatory agencies and pharmaceutical companies in developing, producing and licensing vaccines for the upcoming influenza season.

https://bit.ly/422WVW7

**Looking ahead**

7 April. World Health Day 2025: healthy beginnings, hopeful futures. https://bit.ly/3FBkwV8

7–11 April. Intergovernmental Negotiating Body 14 pandemic agreement negotiations. WHO Headquarters and online. https://bit.ly/4hxKeXM

19–27 May. Seventy-eighth World Health Assembly. Geneva, Switzerland. https://bit.ly/3FE0auj

